# Protein nanoparticles induce the activation of voltage-dependent non-selective ion channels to modulate biological osmotic pressure in cytotoxic cerebral edema

**DOI:** 10.3389/fphar.2024.1361733

**Published:** 2024-07-26

**Authors:** Wei Fan, Liming Liu, Yuxuan Yin, Jiayi Zhang, Zhaoshun Qiu, Jun Guo, Guangming Li

**Affiliations:** ^1^ Department of Anesthesiology, Huaian First People’s Hospital, Nanjing Medical University, Huaian, China; ^2^ School of Medicine and Holistic Integrative Medicine, Nanjing University of Chinese Medicine, Nanjing, China

**Keywords:** astrocyte edema, protein nanoparticles, osmosis, voltage-dependent nonselective ion channels, multi-targeting

## Abstract

**Introduction:**

Cytotoxic cerebral edema is a serious complication associated with cerebral ischemic stroke and is widely treated using the hypertonic dehydrant. Here, we propose, for the first time, the decrease of intracellular osmosis as a treatment strategy for alleviating cytotoxic cerebral edema.

**Methods:**

We established a fluorescence resonance energy transfer-based intermediate filament tension probe for the study and *in situ* evaluation of osmotic gradients, which were examined in real-time in living cells from primary cultures as well as cell lines. The MCAO rat model was used to confirm our therapy of cerebral edema.

**Results:**

Depolymerization of microfilaments/microtubules and the production of NLRP3 inflammasome resulted in an abundance of protein nanoparticles (PNs) in the glutamate-induced swelling of astrocytes. PNs induced changes in membrane potential and intracellular second messengers, thereby contributing to hyper-osmosis and the resultant astrocyte swelling via the activation of voltage-dependent nonselective ion channels. Therefore, multiple inhibitors of PNs, sodium and chloride ion channels were screened as compound combinations, based on a decrease in cell osmosis and astrocyte swelling, which was followed by further confirmation of the effectiveness of the compound combination against alleviated cerebral edema after ischemia.

**Discussion:**

The present study proposes new pathological mechanisms underlying “electrophysiology-biochemical signal-osmotic tension,” which are responsible for cascade regulation in cerebral edema. It also explores various compound combinations as a potential treatment strategy for cerebral edema, which act by multi-targeting intracellular PNs and voltage-dependent nonselective ion flux to reduce astrocyte osmosis.

## 1 Introduction

Cerebral edema is a serious complication of ischemic stroke and cerebral hemorrhage, with high incidence, disability and fatality rates. In general, the fatal cases are associated with the intracranial hypertension-induced nerve cell deformation and neurological impairment. Alleviating cerebral edema is an effective strategy for the clinical treatment of ischemic brain injury, and has been shown to prevent disease progression and improve prognosis (Bar and Biller, 2018; Wu et al., 2018; Gu et al., 2022; Han et al., 2022). Cytotoxic edema occurs in the primary stage of cerebral edema and is mainly presented as astrocyte swelling (Halstead and Geocadin, 2019; Jha et al., 2019). As the most abundant cells in the central nervous system, the astrocytes stimulated by ischemia and hypoxia show considerable swelling, leading to cerebral injury and difficult prognosis for stroke patients (Lee et al., 2022). Multiple lines of evidence suggest that the excessive accumulation of the extracellular neurotransmitter glutamate can activate intracellular calcium, leading to the abnormal accumulation of intracellular cations and resulting in astrocyte edema (Mahmoud et al., 2019; Song et al., 2020; van Putten et al., 2021; Menyhárt et al., 2022). Therefore, osmotic pressure (OP) disequilibrium leads to the influx of ions and water, which is the predominant driving force of cytotoxic brain edema. The most common treatment for cerebral edema is osmotic therapy, with mannitol serving as the hypertonic agent. However, repeated treatments would result in an accumulation of mannitol in the injured brain tissue, resulting in the reflux of interstitial fluid and edema recurrence (Deng et al., 2016; Papagianni et al., 2018; Cook et al., 2020). Hence, we propose that a decrease in astrocyte osmosis can help alleviate cytotoxic edema.

Previous studies on cerebral edema pathogenesis and treatment focus on the aquaporins (AQPs). AQP4, the predominant AQP, has high protein expression during cytotoxic cerebral edema and has been shown to be involved in cerebral water balance and glial scar. However, the transmembrane OP gradients are prerequisites for the AQP-induced water flux. Ion channels determine the ion disequilibrium between the two sides of the cell membrane. Sulfonylurea receptor 1-transient receptor potential melastatin subfamily member 4 (SUR1-TRPM4) non-selective cation channel connects with AQP4 to form complex that enhances ion-water coupling osmosis, thereby facilitating astrocyte swelling (Stokum et al., 2018; Jha et al., 2021). Glyburide (SUR1-TRPM4 inhibitor) was developed as a targeted drug for treating edema (Tan et al., 2014; King et al., 2018; Nakayama et al., 2018); however, its effectiveness has since been challenged. In a double-blind and placebo-controlled trial in the United States, glyburide was tolerated in patients with cerebral infarction, although there was no significant difference in prognosis (Sheth et al., 2016). SUR1-TRPM is unlikely to be the only channel determining cytotoxic edema. Chloride channel TMEM16A and hyperpolarization-activated cyclic nucleotide-gated (HCN) channel are also involved in brain infarct and neurological deficits after ischemic stroke (Liu et al., 2019; Park et al., 2019). Cystic fibrosis transmembrane conductance regulator (CFTR) is a chloride ion channel involved in edema formation, and its activity is enhanced by the increased cAMP levels (Kunzelmann and Mehta, 2013a; Pankonien et al., 2022).

Our previous studies have shown that protein nanoparticles (PNs) play a crucial role in regulating cell osmosis (Zheng et al., 2021; Qian et al., 2022). The depolymerization of microfilaments (MF) and microtubules (MT) stimulate the production of PNs, thereby facilitating astrocyte swelling (Zhang et al., 2019). This raises a few questions. How does the low concentration of PNs trigger hyper-osmosis in living cells? What is the underlying electrochemical and mechanical mechanism associated with the selective opening of ion channels? We used a fluorescence resonance energy transfer (FRET)-based intermediate filament (IF) tension probe for this study. Its tension is linearly related to the transmembrane OP in living cells (Zheng et al., 2022a). This probe can be used for the real-time *in situ* monitoring of astrocyte osmosis. The present study identifies the voltage-dependent ion channels, which are co-activated by PN-induced membrane potential and intracellular chemical signals. Inhibitors specific to PNs production, as well as various non-selective ion channels are screened. Multi-targeted blocking and balancing of the intracellular osmosis could be new therapeutic strategies for the treatment of cerebral edema.

## 2 Materials and methods

### 2.1 Cell culture and reagents

Suckling mice aged 24–48 h were used to extract primary cells. Cerebral cortex tissues were stripped and minced, followed by digestion with trypsin. The digestion was terminated by the addition of 20% FBS in the DMEM medium. Cells were cultured in a flask kept inside an incubator maintained at 37°C and 5% CO_2_. After 48 h, the primary astrocytes were digested with 0.125% Trypsin EGTA. The digestion was terminated by the addition of 20% FBS-DMEM medium. The cells were centrifuged and resuspended, followed by their inoculation in a new culture flask to allow for the growth of adherent cells for further experiments. Astrocyte U87 cells were cultured at 37°C, in 5% CO_2_ with Dulbecco’s Modified Eagle’s Medium (DMEM; Gibco, Grand Island, NY, USA) containing 10% fetal bovine serum (FBS; Gibco), 100 μg/mL penicillin, and 100 μg/mL streptomycin (Gibco).

The ischemia reperfusion model of astrocyte was cultured *in vitro* by oxygen glucose deprivation/reperfusion method. The cells were cultured in sugar-free DMEM medium in an incubator maintained at 37°C in an atmosphere of 95% N_2_ and 5% CO_2_ for 4 h. This was followed by further culture of the cells in normal DMEM medium for the subsequent experiments.

### 2.2 GFAP FRET probe construction and transfection

The FRET-based tension probes of glial fibrillary acidic protein (GFAP)-cpst-GFAP (GcpG) were designed and constructed as described in previous reports (Zhang et al., 2019; Zheng et al., 2022a). The integrity of all expression constructs was confirmed by DNA sequencing. Plasmids encoding the FRET sensor were transfected into cells with FuGENE® 6 transfection reagent (Roche Diagnostics, Basel, Switzerland) and Opti-MEM™ media (Invitrogen, Carlsbad, CA, United States), according to manufacturers’ instructions. The samples were analyzed using the following excitation lasers and emission filters: Cyan-458-nm laser and 447/60 bandpass filter; Yellow-514-nm laser and 580/23 bandpass filter. Cells were sorted with the simultaneous detection of cyan and yellow emission wavelengths.

### 2.3 cpstFRET analyses

The efficiency of FRET is determined by the distance and the dipole angular orientation between the donor/CFP and the acceptor/YFP. The cells were imaged using a confocal microscope (SP5; Leica, Wetzlar, Germany) equipped with a ×63 oil-immersion objective lens. The donor and acceptor were observed under 458 nm and 514 nm argon lasers, respectively. CFP/FRET ratios were calculated using the equation 1/E = Cerulean (donor)/Venus (acceptor) (Hu et al., 2022).

### 2.4 Measurement of cytoplasmic OP and count rate of protein particles

Astrocyte U87 cells were cultivated in 90-mm Petri dishes. When the cell proliferation reached about 95%, the medium was discarded, the cells were washed twice with isotonic Hepes buffer, and appropriate experimental stimulation was added. After 24 h, the drug-containing culture medium was aspirated, digested by typsin and washed, and the cell suspension was collected into 1.5 mL microcentrifuge tube. Centrifuge at 1,000 *g* at 4°C for 5 min and discard supernatant. Following ultrasonification (75% amplitude, five times, 5 s) (Sonics and Materials, Connecticut, CT, United States) and ultracentrifugation (20,000 g, 1 h, room temperature), 50 µL of the supernatant solution (cytoplasm) was transferred into 0.5-mL test tubes. The cytoplasmic OP was measured using the Osmomat 3000 Freezing Point Osmometer (Gonotec, Berlin, Germany) The diluted supernatant was used to assess the count rate (kilocycles per second, kcps) of cytoplasmic nanoparticles (NanoSight NS300; Malvern Instruments, Malvern, United Kingdom).

### 2.5 Ca^2+^, Na^+^ and Cl^−^ fluorescent imaging to detect the intracellular ion content

Calcium, chloridion and sodium fluorescent imaging was performed using the dyes Fluo-4 a.m. (excitation at 494 nm and emission at 516 nm, purchased from Abcam, Cambridge, England), N-(ethoxycarbonylmethyl)-6-methoxyquinolinium bromide (MQAE, excitation at 355 nm and emission at 460 nm, purchased from Beyotime, Shanghai, China), and Enhanced NaTrium Green (ENG)-2 a.m. (excitation at 488–525 nm and emission at 545 nm, purchased from New Research Biosciences, Xian, China), respectively. After the specific treatment, the cell cultures were examined by confocal microscopy to confirm comparable fluorescence intensities.

### 2.6 DAG and cAMP content determination

Cell cytoplasm supernatants were obtained and processed according to the instructions of the human diglyceride (DAG) and cAMP ELISA kit provided by Jiangsu Enzyme Immunoassay Industry Co., Ltd. and Nanjing Yifeixue Biotechnology Co., Ltd, respectively.

### 2.7 3D cell imaging

Cells were inoculated into a glass plate with a diameter of 35 mm. The cells adhered to the wall and were allowed to grow until they reached 60%–70% confluence. They were subsequently treated with drugs and maintained in an incubator at 37°C in an atmosphere of 95% O_2_ and 5% CO_2_. After 12 h, the cells were analyzed using ×3 objective 60D tomographic scanning microscope (Nanolive SA, Tolochenaz, Switzerland) and STEVE software (Nanolive SA, Tolochenaz, Switzerland) to determine the changes in their morphology.

### 2.8 Electrophysiology

Whole-cell patch recordings were conducted as previously described (Pankonien et al., 2022). Briefly, the cells were transferred to a submersion-type recording chamber and perfused (1∼2 mL/min) with an extracellular solution composed of 140 mM NaCl, 4 mM KCl, 2 mM CaCl2, 1 mM MgCl2, 10 mM glucose, and 10 mM HEPES, with the pH adjusted to 7.4 using NaOH. The intracellular solution for membrane potential recordings was composed of 130 mM K-gluconate, 10 mM NaCl, 2 mM MgCl_2_, 2 mM CaCl_2_, 10 mM EGTA, 2 mM Na_2_ATP, pH adjusted to 7.2 with KOH. The average resistance of the recording pipettes was 2∼6 MΩ, when filled with internal solution. Recordings were performed using a MultiClamp 700B amplifier and a Digidata 1550B interface (Molecular Devices, CA, United States) at room temperature (24∼25°C).

Whole-cell voltage clamp recordings were performed on astrocyte cells. Cells were spread at an appropriate density on polylysine-coated cell slides, placed in a bath on the stage of an inverted microscope, and perfused with the desired solution. The electro physiological results were performed using a Multiclamp 700B amplifier at a data-sampling rate of 2 kHz with a Digidata 1550B Digitizer controlled using PClamp 10.6 software (Molecular Devices, Sunnyvale, CA, United States). A glass electrode (outer diameter 1.5 mm; internal diameter 0.86 mm; World Precision Instruments, Sarasota, FL, United States) using a drawing instrument (P-1000; Narishige Scientific Instrument Lab., Tokyo, Japan) were drawn with resistance values of 2–5 MΩ after filling the inner fluid, and all experiments were performed at room temperature (23°C–25°C). Adjustments for capacitance compensation and series resistance compensation were made before recording. Series resistance was routinely compensated by 60%–80%. If the recording channel was unstable during the experiment (the series resistance increased by more than 15% at the end of the experiment), the cell was discarded from the data analysis. The Current trace diagram was performed in Graphpad Prism 7.

### 2.9 Establishment of the rat MCAO model, assessment of cerebral ischemic volume and determination of brain water content

Rats were anesthetized using an intraperitoneal injection of 2% sodium pentobarbital (30 mg/kg). Body temperature was monitored and maintained at 36.5°C–37.5°C. Briefly, after making an incision in the midline skin, the right common carotid artery, external carotid artery (ECA), and internal carotid artery (ICA) were exposed. Subsequently, a 6–0 nylon monofilament with a rounded tip was inserted into the right ICA through the broken end of the ECA, in order to block the origin of the middle cerebral artery (MCA). Cerebral ischemia through the intraluminal suture was maintained for 60 min. It was followed by the removal of the monofilament and reperfusion. After 24 h of middle cerebral artery occlusion (MCAO), the brains were obtained by decapitation. This experiment was approved by the ethics committee of the Affiliated Huaian First People’s Hospital of Nanjing Medical University.

The infarct volume was evaluated at 24 h after MCAO using 2, 3, 5-triphenyltetrazolium chloride (TTC) staining. The volumes of contralateral and non-infarcted tissue of the ipsilateral hemispheres were outlined and measured with ImageJ software (NIH). The formula used for calculating infract volume is given below: Infract volume = (volume of contralateral—volume of non-ischemic ipsilateral)/2*(volume of contralateral) *100% (Zhou et al., 2021). The brains were harvested from different groups of rats and weighed (wet weight). They were dried for 2 days in a 70°C incubator, which was followed by the determination of their dry weight. The water content was calculated using the formula: Water content = (wet weight – dry weight)/wet weight * 100%.

### 2.10 Serum biochemical assays

The blood obtained from different groups was placed at room temperature for 1 h, followed by centrifugation at 3,000 rpm/min for 10 min in order to obtain serum. The levels of glutamic-pyruvic transaminase (ALT), glutamic oxalacetic transaminase (AST), alkaline phosphatase (ALP), blood urea nitrogen (BUN) and creatinine (CRE) in serum were measured using specific kits (Nanjing Jiancheng Bioengineering Institute, China) in accordance with the manufacturer’s instructions.

### 2.11 Data analysis

Data analysis was conducted using the statistical program Graph Pad Prism 7. More than three biological replicates were presented as mean ± SEM. Unpaired *t*-test (two-tailed *p*-value) was used to determine the statistical significance (*p* < 0.05 was considered to be statistically significant).

## 3 Results

### 3.1 Inhibition of intracellular PNs via inflammasome suppression and MF/MT stabilization can decrease osmolarity and astrocyte swelling

**FIGURE 1:**
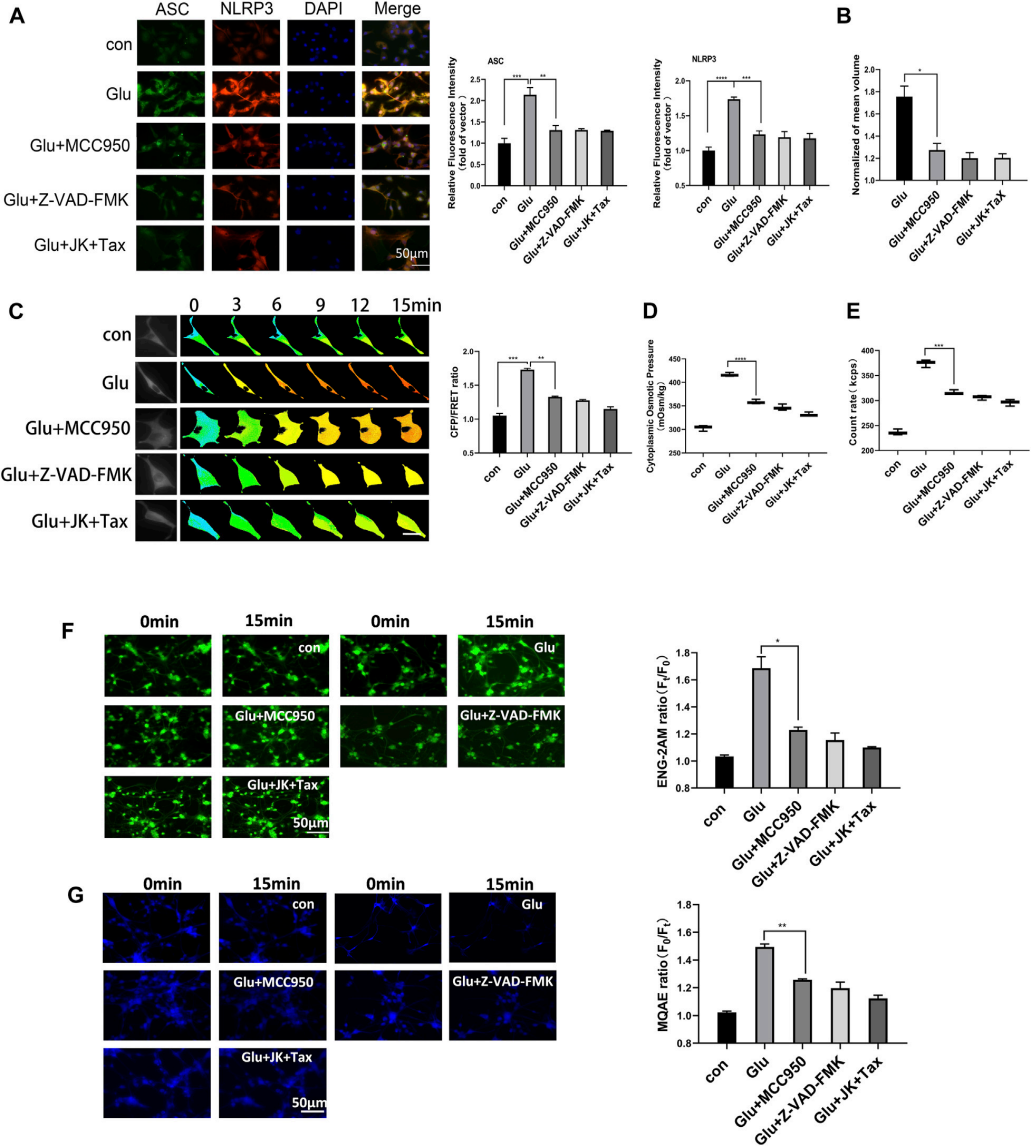
NLRP3 inflammasome and MF/MT depolymerization induced PNs, resulting in hyperosmosis. **(A)** Immunofluorescence image and relative fluorescent intensity analysis of ASC and NLRP3 in primary astrocytes under glutamate (Glu, 2 mM) co-treated with MCC950 (NLRP3 specific inhibitor, 1 μM), Z-VAD-FMK (caspase-1 pan inhibitor, 20 μM) and JK (microfilament stabilizer, 1 μM)-TAX (microtubule stabilizer, 10 μM). **(B)** Normalized cell volume of primary astrocytes, as detected by the 3D Cell Imaging. **(C)** Representative images and mean values of normalized CFP/FRET ratios of IF tension using GFAP FRET-based tension probe in primary astrocytes. Calibration bar was set from 0.1–1.5. **(D)** Cytoplasmic OP values of astrocyte cell line U87 were measured using a freezing point osmometer. **(E)** Count rate of PNs in U87 cells. **(F)** Na^+^ imaging micrographs and traces of relative ENG fluorescence intensity (Ft/F0) of primary astrocytes. **(G)** Cl^−^ imaging micrographs and traces of relative MQAE fluorescence intensity (F0/Ft) of primary astrocytes. The increase in intracellular Cl^−^ levels results in the decrease of MQAE fluorescence value instead. The values represent mean of ≥3 experiments ±SEM. Values marked with asterisks represent statistically significant differences.

Cerebral ischemic injury induces extracellular glutamate accumulation, further facilitating intercellular accumulation of cations and neurocyte swelling (Mahmoud et al., 2019; van Putten et al., 2021). We exposed primary astrocytes and the astrocyte cell line to medium containing glutamate (2 mM). Our previous research demonstrated that glutamate stimulated MF and MT depolymerization in astrocytes, resulting in the production of actin and tubulin monomers. These monomers function as intracellular PNs and promote hyper-osmosis and astrocyte swelling (Zheng et al., 2021). We hypothesize that protein nanoparticle-induced osmotic pressure (PN-OP) plays a significant role in astrocyte edema. In this study, we found that glutamate induced an increase in the levels of NLRP3 and ASC. MCC950 (NLRP3 specific inhibitor) or Z-VAD-FMK (caspase-1 pan inhibitor) effectively rescued the glutamate-induced ASC and NLRP3 in the primary astrocytes (Figure 1A). Interestingly, MF stabilizer jasplakinolide (JK) and MT stabilizer taxol (TAX) co-treatments also reduced ASC and NLRP3, suggesting that MF and MT stabilization relieved the NLRP3 inflammatory effects in response to glutamate stimuli. In addition, MCC950, Z-VAD-FMK, JK-TAX treatments significantly decreased glutamate-induced astrocyte swelling, as shown by 3D cell imaging (Figure 1B). Thus, we postulate that NLRP3 inflammasome is another intracellular PNs involved in astrocytic OP regulation. To investigate this theory, a glial fibrillary acidic protein (GFAP) FRET-based tension probe was applied in primary astrocytes to monitor IF tension in real-time, as it has been shown to be an effective probe in evaluating the transmembrane OP effects in living cells (Qian et al., 2022). MCC950, Z-VAD-FMK, and JK-TAX treatments significantly reduced glutamate-induced IF tension (Figure 1C). In parallel, these inhibitors decreased the cytoplasmic OP and the amount of intracellular PNs, as detected by the freezing point osmometer and nanoparticle tracking analyzer, respectively (Figures 1D,E). Meanwhile, the fluorescent imaging results showed that NLRP3 and MF/MT inhibition decreased the sodium and chloride ion levels in primary astrocytes (Figures 1F,G), which are closely associated with intracellular osmosis. Similar results were also observed in the astrocyte cell line U87 (Supplementary Figure S1). Taken together, the cell volume, IF tension, cytoplasmic OP and the intracellular ion levels showed variations which were consistent with regard to the inhibition of PNs. These findings suggest that NLRP3 inflammasome production and MF/MT depolymerization are the two main sources of intracellular PNs, which upregulate cell osmosis and promote astrocyte swelling.

### 3.2 Intracellular PNs trigger membrane potential changes and an increase in the levels of second messenger

**FIGURE 2:**
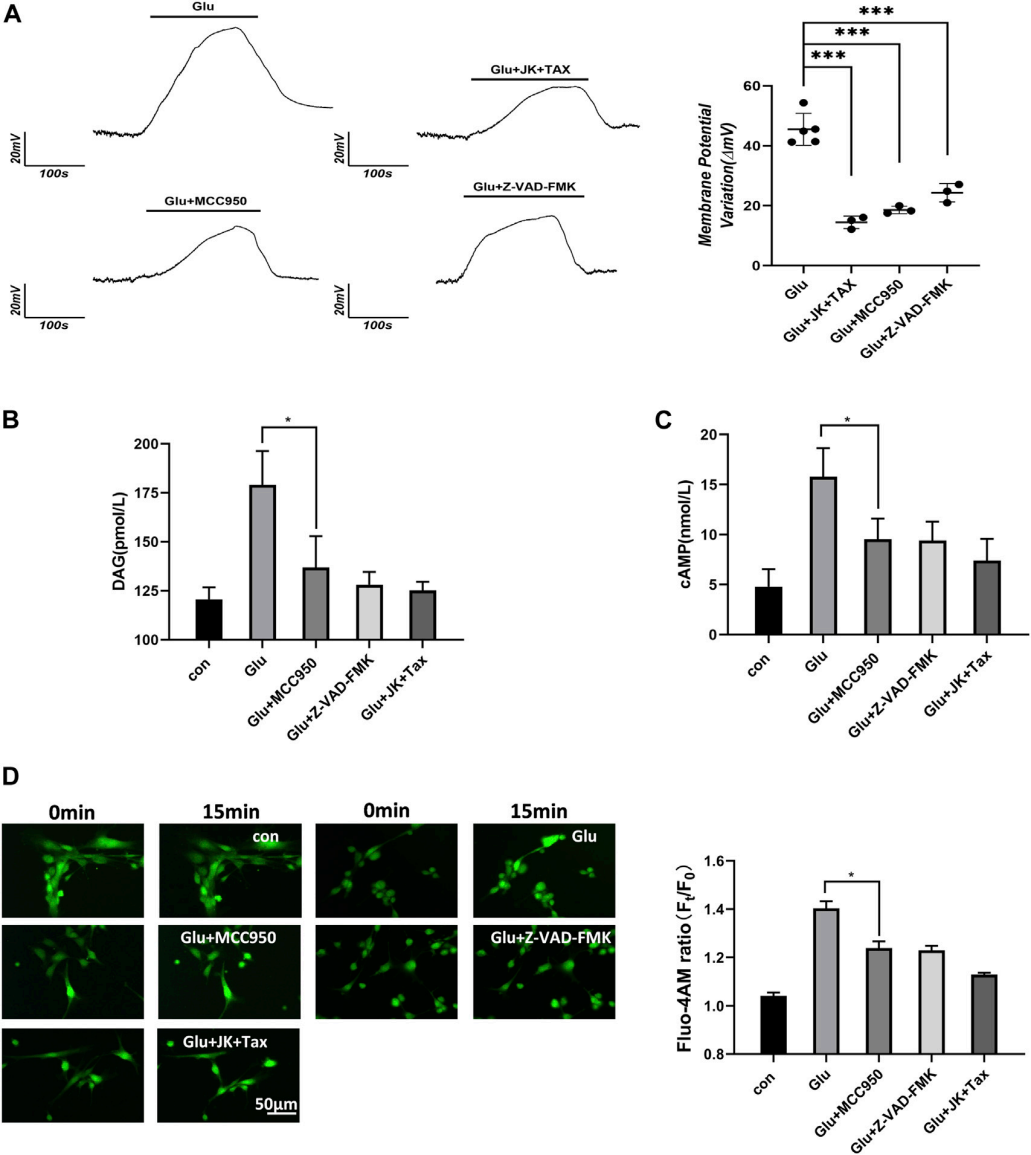
PNs induce membrane potential changes and increase the level of second messenger. **(A)** Membrane potential of primary astrocytes was measured using the whole-cell current clamp. **(B)** DAG was detected from primary astrocytes using ELISA. **(C)** cAMP was detected from primary astrocytes using ELISA. **(D)** Ca^2+^ imaging micrographs and traces of relative Fluo-4 fluorescence intensity (Ft/F0) of primary astrocytes.

According to the Donnan effect, intracellular PNs can absorb cations and promote the re-arrangement of free ions near the plasma membrane, leading to changes in membrane potential (Nguyen and Kurtz, 2006; Lang et al., 2014; Yu et al., 2021). Membrane potential was measured by the current clamp of primary astrocytes and showed depolarization corresponding to the glutamate stimuli. However, a decrease in the intracellular PNs through the MF/MT stabilization and NLRP3 inhibition resulted in a significant recovery of the depolarized astrocytes (Figure 2A).

Astrocytes respond to external stimuli by activating cell surface receptors that increase the intracellular levels of secondary messengers, including diacylglycerol (DAG), cyclic adenosine phosphate (cAMP) and Ca^2+^. Increased Ca^2+^ concentration in cells can activate adenylate cyclase 3, leading to the enhanced synthesis of intracellular cAMP. We performed the DAG and cAMP ELISA assays, and the Flou-4 a.m. Ca^2+^ fluorescent imaging in primary astrocytes. Glutamate stimulated an increase in the levels of the second messengers, including diacylglycerol (DAG), cAMP and Ca^2+^. However, when PN production was blocked by the NLRP3 inhibitor MCC950, caspase-1 inhibitor Z-VAD-FMK, or MF/MT stabilizers JK/TAX, it also caused a decrease in the levels of second messengers (Figures 2B–D). These results indicate that intracellular PNs can trigger changes in the membrane potential and induce second messengers during astrocyte swelling.

### 3.3 Voltage and second messenger-dependent ion channels involved in astrocyte swelling

**FIGURE 3:**
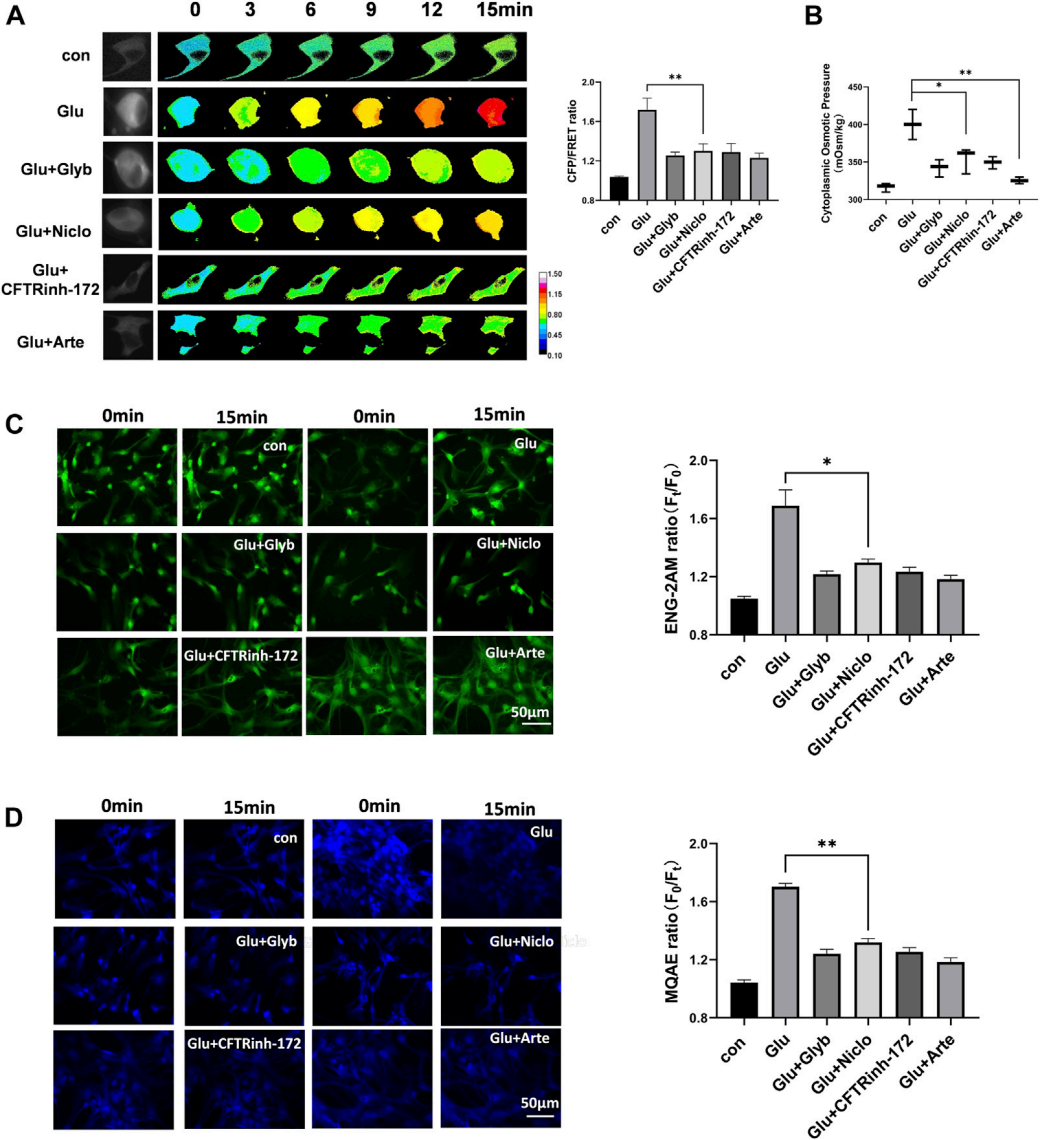
Voltage-dependent ion channels are involved in glutamate-induced astrocyte hyperosmosis. **(A)** Representative images and mean values of normalized CFP/FRET ratios of IF tension using GFAP FRET-based tension probe in primary astrocytes under different treatments of glutamate and Glyb (TRPM4 inhibitor, 10 μM), Niclo (TMEM16A inhibitor, 1 μM), CFTRinh-172 (CFTR inhibitor, 1 μM) or Arte (HCN inhibitor, 10 μM). Calibration bar was set from 0.1–1.5. **(B)** Cytoplasmic OP values of astrocyte cell line U87. **(C)** Na^+^ imaging micrographs and traces of relative ENG fluorescence intensity (Ft/F0) of primary astrocytes. **(D)** Cl^−^ imaging micrographs and traces of relative MQAE fluorescence intensity (F0/Ft) of primary astrocytes. The values represent mean of ≥3 experiments ±SEM. Values marked with asterisks represent statistically significant differences.

Corresponding to changes in the membrane potential and the levels of various second messengers, we predict the activation of voltage-dependent ion channels. Ca^2+^-activated TRPM4 channel has been reported to predominantly control the massive Na^+^ influx involved in edema progression (Stokum et al., 2018). Furthermore, Ca^2+^-activated TMEM16A (also known as anoctamin-1) function as Cl^−^ channels, and open in response to cerebral infarction (Liu et al., 2019). To investigate the involvement of these ion channels in astrocyte osmosis, primary astrocytes induced by glutamate and the U87 cell line were individually exposed to glyburide (Glyb, TRPM4 inhibitor) and niclosamide (Niclo, TMEM16A inhibitor). Results showed that the two inhibitors effectively attenuated the osmotic effects, including IF tension (Figure 3A), cytoplasmic OP (Figure 3B), and the Na^+^ and Cl^−^ levels in astrocytes (Figures 3C,D, Supplementary Figure S2).

Another second messenger of cAMP promotes voltage-dependent activation of hyperpolarization and cyclic nucleotide gated (HCN) cation channel involved in the Na^+^/K^+^ electrochemical gradient flow (Benarroch, 2013). Cystic fibrosis transmembrane conductance regulator (CFTR) is a chloride ion channel, and its activity is enhanced by an increase in the cAMP levels (Kunzelmann and Mehta, 2013b; Weidenfeld and Kuebler, 2017). We found that artemisinin (Arte, HCN inhibitor) and CFTRinh-172 (CFTR inhibitor) caused a significant suppression of glutamate-induced IF tension, cytoplasmic OP, and the Cl^−^ and Na^+^ content in both primary astrocytes and the U87 cells (Figure 3 and Supplementary Figure S2). Further, Arte could effectively reduce the HCN current (Supplementary Figure S3). Taken together, the glutamate stimuli simultaneously induced various ion channels, which were dependent on both membrane potential and second messengers. These ion channels could federatively promote the ion influx-mediated astrocyte swelling.

### 3.4 Screening of compound combination screened by reducing cell osmosis against astrocyte swelling

**FIGURE 4:**
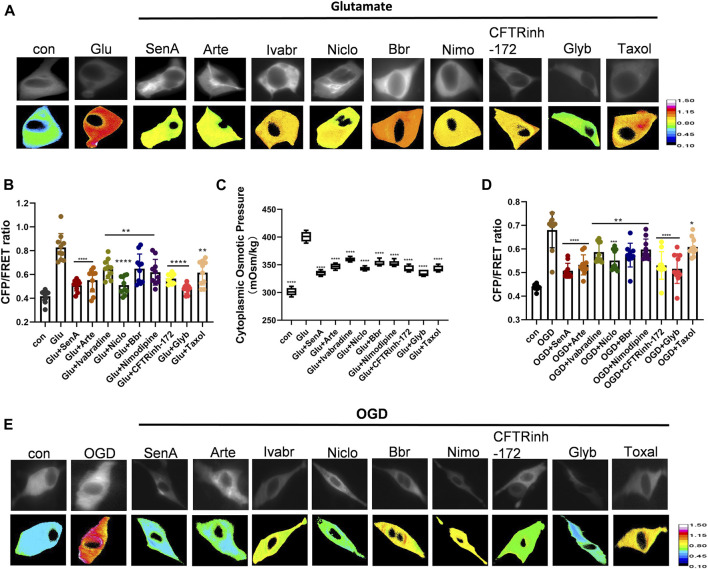
Screening of compounds against astrocyte swelling. **(A), (B)** Representative images and mean values of normalized CFP/FRET ratios of IF tension using GFAP FRET-based tension probe in astrocyte cell line U87 under different treatments of glutamate and SenA (microfilament stabilizer 50 μM), Arte (HCN inhibitor, 10 μM), Ivabradine (HCN inhibitor, 50 μM), Niclo (TMEM16A inhibitor, 1 μM), Bbr (TMEM16A inhibitor, 1 μM), Nimodipine (VGCC Ca^2+^ channel inhibitor, 30 μM), CFTRinh-172 (CFTR inhibitor, 1 μM), Glyb (TRPM4 inhibitor, 10 μM) and Taxol (microtubule stabilizer, 10 μM**)**. **(C)** Cytoplasmic OP values of astrocyte cell line U87. **(D), (E)** Representative images and mean values of normalized CFP/FRET ratios of IF tension in primary astrocyte OGD model individually treated with different compounds. Calibration bar was set from 0.1–1.5. The values represent mean of ≥3 experiments ±SEM. Values marked with asterisks represent statistically significant differences.

As intracellular PNs and various ion channels regulate astrocyte swelling, we next screened for potential compounds against edema. Shingshot (SSH) inhibitor Sennoside A (SenA) can inhibit cofilin dephosphorylation to stabilize MF (Lee et al., 2017). Taxol is widely used as a MT stabilizer (Yang and Horwitz, 2017). Nimodipine is a voltage-gated L-type calcium channel inhibitor that blocks Ca^2+^ influx to maintain intracellular calcium homeostasis (Carlson et al., 2020). Glyburide (Glyb) is SUR1-TRPM4 channel inhibitor (King et al., 2018; Nakayama et al., 2018), while Niclosamide (Niclo) and Benzbromarone (Bbr) function as TMEM16A inhibitors (Miner et al., 2019; Dwivedi et al., 2023). Artemisinin (Arte) and Ivabradine are specific blockers of HCN channel (Ide et al., 2019), and CFTRinh-172 is a specific inhibitor of CFTR channel (Melis et al., 2014). The glutamate-induced astrocyte U87 cells and oxygen-glucose deprivation (OGD)-induced primary astrocytes were individually treated with the above-mentioned inhibitors. All of them attenuated the glutamate-induced effects on IF tension and cytoplasmic OP; with SenA, Glyb, Niclo, Arte and CFTRinh-172 showing high efficiencies (Figure 4).

**FIGURE 5:**
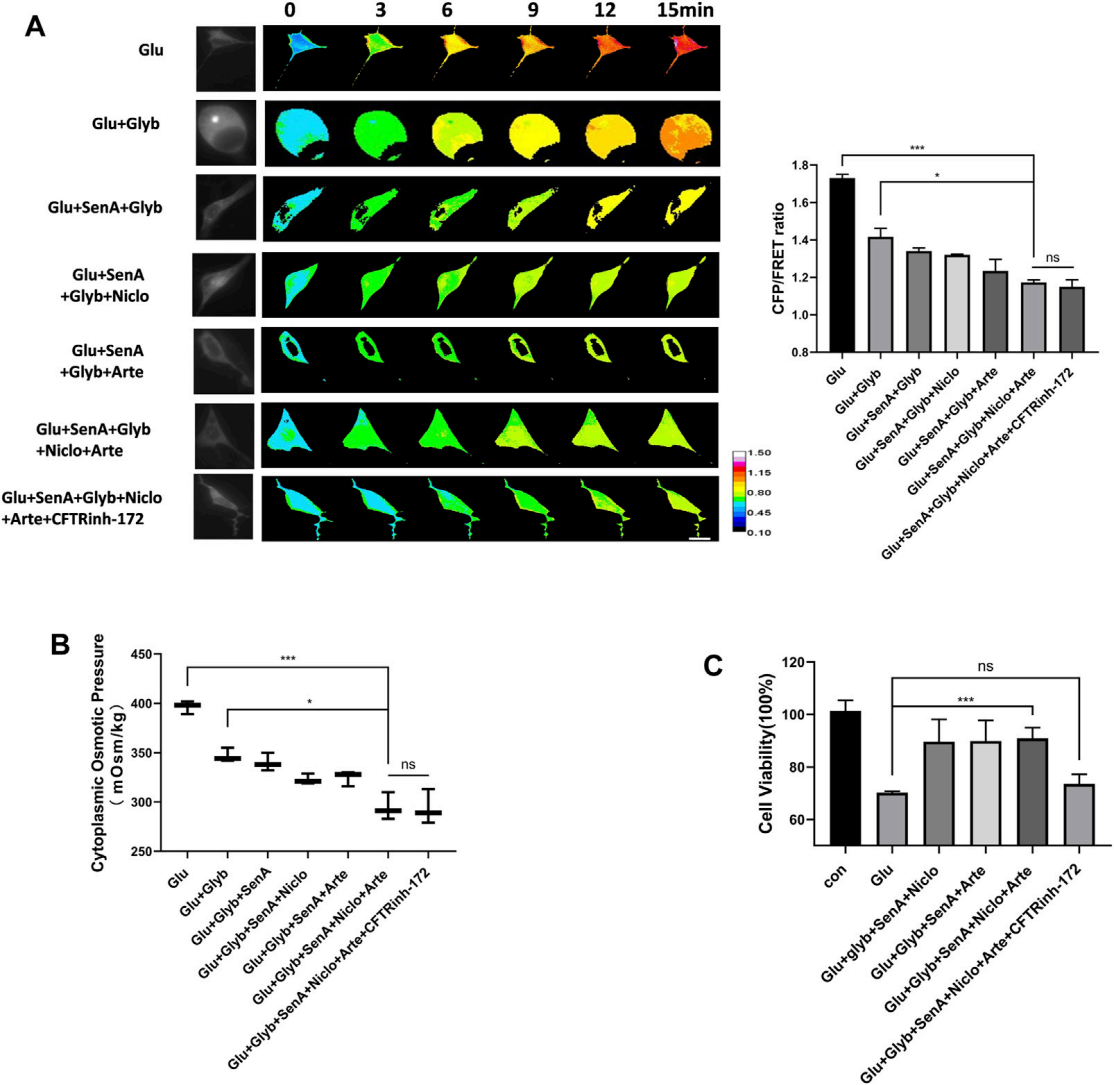
Compound combination effectively alleviates glutamate-induced astrocyte hyperosmosis. **(A)** Representative images and mean values of normalized CFP/FRET ratios of IF tension using GFAP FRET-based tension probe in primary astrocytes treated with different drug combinations. **(B)** Cytoplasmic OP values of astrocyte cell line U87. **(C)** Cell viability of primary astrocytes treated with different drug combinations, as detected by CCK8 assay. The values represent the mean of ≥3 experiments ±SEM. Values marked with asterisks represent statistically significant differences while ns represents no significant difference.

To investigate whether these MF stabilizer and ion channel inhibitors display a combined effect against glutamate-induced primary astrocyte edema, different compound designs were tested for their osmotic effects. Glyb is widely used as a drug against edema; however, Glyb treatment alone failed to ensure complete recovery of the glutamate-induced IF tension (Figure 5A). Combinations of two compounds (SenA and Glyb) and three compounds (SenA, Glyb and Niclo) showed mild effects. Combinations of four and five compounds, containing Glyb/SenA/Niclo/Arte and Glyb/SenA/Niclo/Arte/CFTRinh-172, showed the highest efficacy on the IF tension (Figure 5A). However, including CFTRinh-172 in the combination dramatically reduced cell viability (Figure 5C), and had little impact on cytoplasmic OP (Figure 5B), thereby suggesting that SenA/Glyb/Niclo/Arte is the optimal combination for the treatment of cytotoxic edema.

### 3.5 Drug-combination treatment effectively alleviates brain edema in the MCAO rat model

**FIGURE 6:**
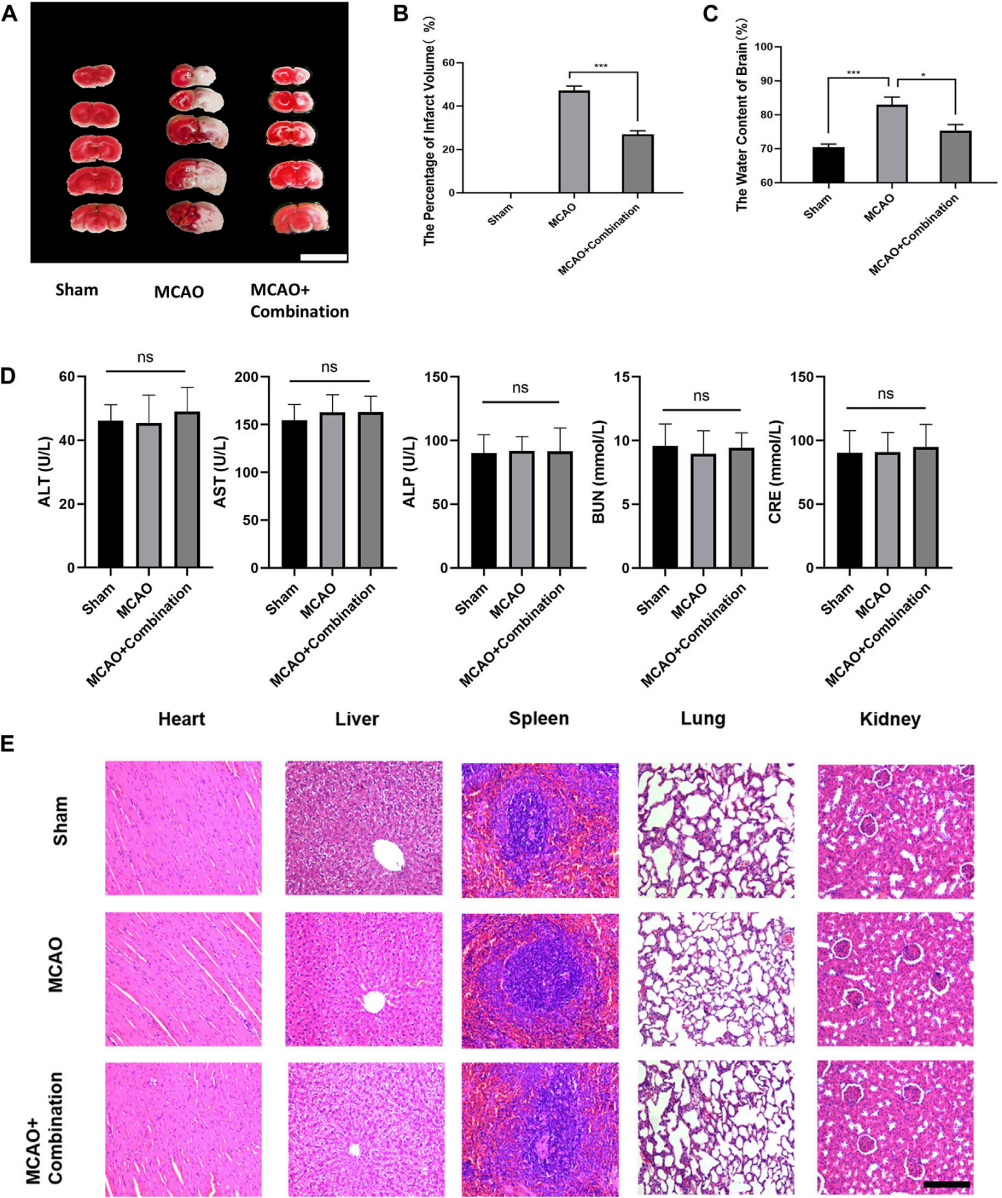
Application of the compound combination in the MCAO rat model to confirm drug efficacy and safety. **(A), (B)** Volumes of contralateral and non-infarcted tissue of the ipsilateral hemispheres were outlined and measured with ImageJ software. The percentage of infarct volume = (volume of contralateral–volume of non-ischemic ipsilateral)/2*(volume of contralateral) *100%. Scare bar: 1 cm **(C)** The water content of brain = (wet weight–dry weight)/wet weight * 100%. **(D)** Serum biochemical assays, **(E)** HE staining of rat tissue sections were performed to determine safety of drug combination. The values represent the mean of 3 experiments ±SEM. Values marked with asterisks represent statistically significant differences while ns indicates no significant difference. Scale bar, 100 μm.

Cerebral ischemia induces extracellular glutamate accumulation, ion disequilibrium and the resultant edema. The MCAO rat model was used to further confirm the efficacy of the above combination compounds *in vivo*. The MCAO group showed a significant increase in the volume of cerebral infarction and water content in brain tissues, while the combination compounds treatment attenuated injuries (Figures 6A–C). In addition, the serum biochemical analysis, which included ALT, AST, ALP, BUN and CRE, and hematoxylin and eosin (HE) staining of tissue section (heart, liver, spleen, lung and kidney), showed no difference among the control, MCAO and drug combination-treated MCAO groups (Figures 6D,E). This confirmed the safety of the drug combination as well as its effective inhibition of cerebral edema.

## 4 Discussion

The present study demonstrates that the simultaneous inhibition of multiple ion channels can effectively attenuate the glutamate-induced Na^+^/Cl^−^ influx and the resultant intracellular hyperosmosis. Intracellular PNs are crucial factors for controlling osmosis in living cells (Kunzelmann and Mehta, 2013a; Park et al., 2019; Zheng et al., 2021; Pankonien et al., 2022). We observed that glutamate simulates the production of abundant intracellular PNs from MF/MT depolymerization and NLRP3 inflammasome, which is accompanied by astrocyte swelling. These PNs carry negative charge and can absorb cations and further drive the changed contents of free ions in and out of the cells. In the present study, we performed whole-cell patch clamp experiments and demonstrated the presence of PN-induced membrane depolarization. Therefore, PNs can induce changes in membrane potential and lead to the rearrangement of intracellular free ions, thereby promoting hyper-osmosis. In contrast to the traditional OP theory, which states that the number of solute particles determine osmosis (Darwish and Lui, 2019; Mitchison, 2019), our new viewpoint proposes that intracellular PNs play an amplified role in promoting hyperosmosis, depending on membrane potential and converting to electrochemical modulation in cerebral edema.

Ion channels are classified into voltage-gated, voltage-dependent and ligand-dependent channels. Membrane potential is not an absolute factor for maintaining the continuous opening of channel, and voltage-gated channels could limit the number of incurrent ions. Multiple ion channels open simultaneously during astrocyte swelling, depending not only on membrane potential, but also on second messengers. Our study showed that the glutamate stimuli induced changes in membrane potential and caused the upregulation of second messengers, including Ca^2+^, cAMP and DAG. The changes in membrane potential are closely associated with the production of second messengers (Figure 2). This occurs via the modulation of Ca^2+^ influx and intracellular Ca^2+^ mobilization, along with the regulation of adenylyl cyclase activity to induce cAMP synthesis (Izumi et al., 1999; Billups et al., 2006; Albarrán et al., 2013). Therefore, second messengers, as intercellular chemical signals, are associated with the synergistic effects on membrane potential changes, thereby leading to the continuous opening of voltage-dependent non-selective ion channels.

Large scale ion influx in the course of astrocyte swelling and cerebral edema enhances the intracellular OP and results in the rapid influx of water. The cation influx was the result of the opening of TRPM4 and HCN channels in response to intracellular Ca^2+^ and cAMP signals (Nilius et al., 2005; Cho et al., 2015; Saponaro et al., 2021). The chloride ions resulted from the opening of chloride channel TMEM16A in response to high levels of intracellular Ca^2+^ (Dutta et al., 2016; Ji et al., 2019). Notably, the Na^+^ and Cl^−^ channels may have a mutual influence on the intracellular cations and anions. TRPM4 and TMEM16A inhibitors decreased the content of both ions in astrocytes. This finding suggests that Cl^−^ influx is not just a Na^+^-driven posterior influx; rather, Na^+^ and Cl^−^ channels have a synergistic role in the regulation of osmosis and edema.

In this study, we show that PN inhibition cannot antagonize the simultaneous opening of various ion channels, thereby implying that PN-induced electrochemical activity is not the only factor promoting hyper-osmosis. Our previous study also showed that cell osmosis is determined not only by the PNs, but also by ion content. For instance, albumins present in isotonic solutions with different ion components (Na^+^ or K^+^) have variable osmotic effects. Therefore, osmotic re-equilibrium is controlled by PNs as well as the various ion channel-mediated rearrangement of the ionic components. PNs and ion channel inhibitors should be taken in combination in order to block the occurrence and development of cerebral edema via the downregulation of intracellular OP. The compound combinations containing Glyb, SenA, Niclo and Arte can simultaneously reduce intracellular PNs and recover intracellular cation and anion levels via the inhibition of various ion channels. This can ultimately lead to a decrease in the intracellular osmosis in cerebral edema.

The present study proposes a new viewpoint of “biological OP,” which is highly dependent on PN-induced electrochemical activity, the synergistic effects of multiple ion channels, the ionic components and content-driven osmosis. Physical OP supports semi-permeable membrane accessible to flow of ions, indiscriminate ion identity. However, the plasma membrane is permeable and allows selective uptake of ions. The transmembrane ion channels control the ion flow in living cells, depending on their ion selectivity. In the physiological condition, the osmotic equilibrium is maintained by the antagonizing transmembrane permeabilities of K^+^ and Na^+^. However, the adsorption of PNs induce membrane potential, while second messengers activate voltage-dependent ion channels, thereby maintaining the osmotic pressure between the two sides of plasma membrane. Thus, the frequency and duration of the opening of various cation and anion channels involved in regulating “biological OP” (Zheng et al., 2022b), as the term of life activity different from the traditional physical OP. Biological OP is crucial mechanical activity in living cells, as regulator of cell morphology, structure and function. Thus, the osmometer developed on the basis of the traditional van der Hoff theory can only detect ions or colloidal solutions, which has defects in evaluating the mechanical effects of the mixed solution of biological ions and protein particles. New established IF tension probe is able to convert the changes of osmotic tension in living cells into optical signals. The transmembrane osmotic pressure of cells generates tension by pulling the intermediate fiber filaments, and the change of the intermediate fiber tension is linearly correlated with the cell osmotic pressure. Observing the fluorescence resonance energy transfer efficiency can effectively evaluate the change of the osmotic potential energy of living cells in real time.

## 5 Conclusion

In summary, the synergistic regulation of “electrophysiological-biochemical signal-osmotic tension” cascades, which aggravate intracellular osmosis and further induce astrocyte swelling, controls the occurrence and development of cytotoxic brain edema. Therefore, based on a number of regulatory factors including PN production and ion channels, we propose a new therapeutic strategy of multi-target blocking and reestablishment of osmotic equilibrium in cells. This provides the pharmacological mechanism for the use of drug combinations for the treatment of cerebral edema.

## Data Availability

The raw data supporting the conclusions of this article will be made available by the authors, without undue reservation.
