# Supplementary material for: Protein nanoparticles induce the activation of voltage-dependent non-selective ion channels to modulate biological osmotic pressure in cytotoxic cerebral edema
Source: Front Pharmacol. 2024 Jul 26;15:1361733. doi: 10.3389/fphar.2024.1361733 (PMC11310023; doi:10.3389/fphar.2024.1361733)
Supplement: Supplementary file 1 [file Image1.pdf]

Supplementary Figures

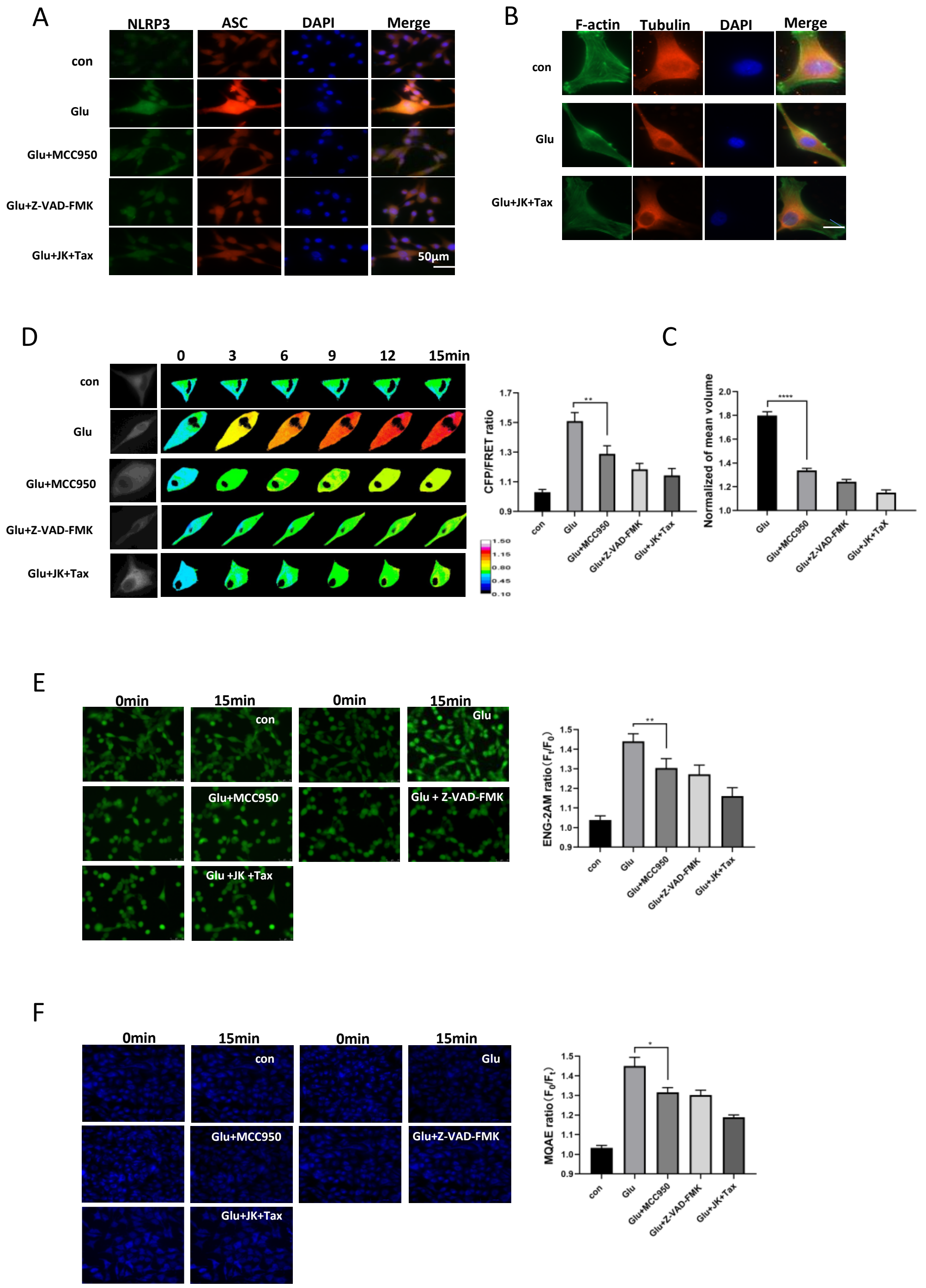

Figure S1. PNs-induced hyperosmosis in astrocyte cell line U87. Immunofluorescence image of ASC and NLRP3 **(A),** F-actin and Tubulin **(B). (C)** Normalized cell volume detected by the 3D Cell Imaging. **(D)** Representative images and mean values of normalized CFP/FRET ratios of IF tension Calibration bar was set from 0.1 to 1.5. **(E)** Na^+ imaging micrographs and traces of relative ENG fluorescence intensity (Ft/F0) of primary astrocytes. **(F)** Cl^- imaging micrographs and traces of relative MQAE fluorescence intensity (F0/Ft). Mean of ≥ 3 experiments ± SEM. Values marked with asterisks represent significantly different.

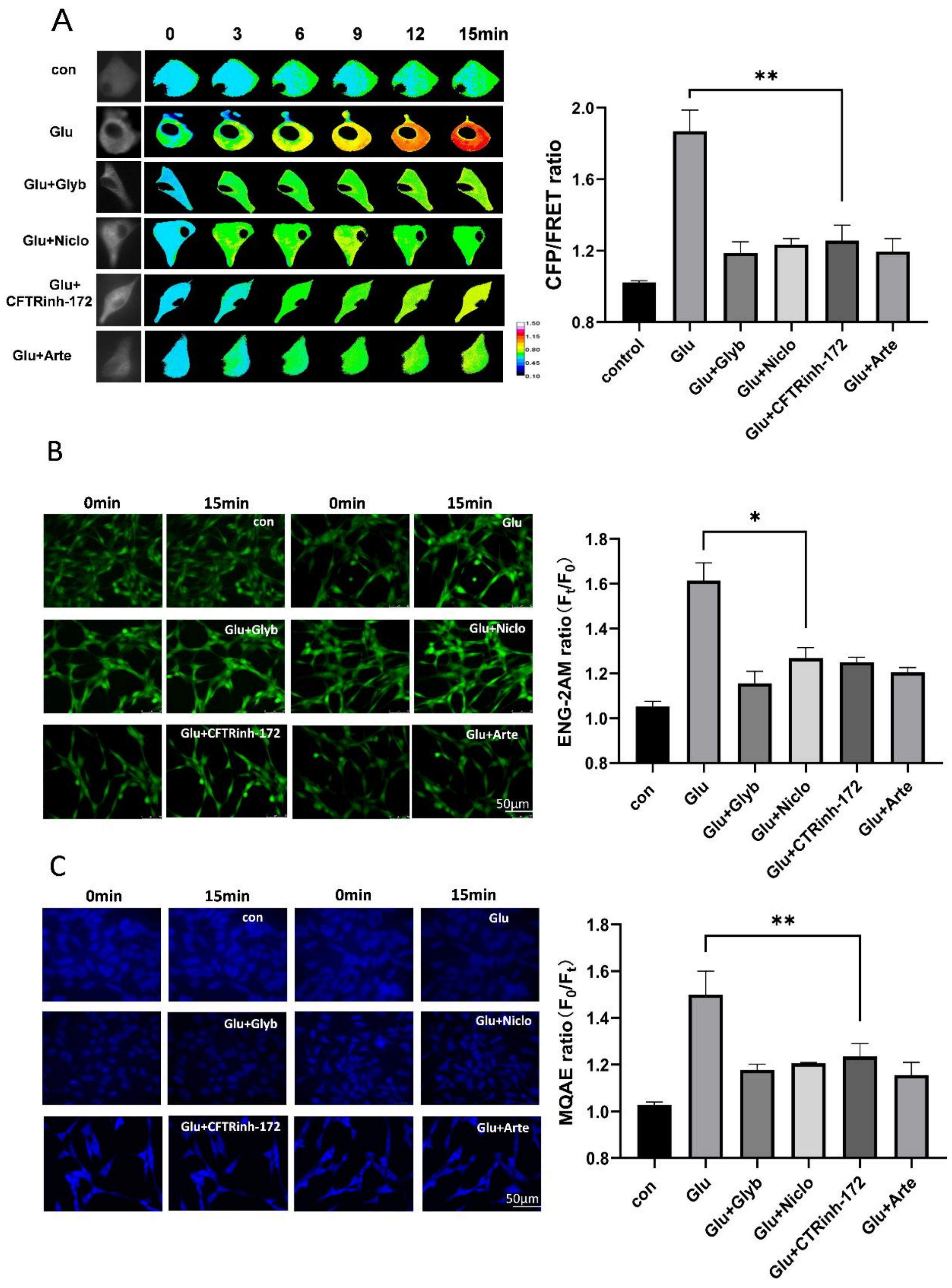

Figure S2. Inhibitors of voltage-dependent ion channels relieved U87 astrocyte hyperosmosis. (A) Representative images and mean values of normalized CFP/FRET ratios of IF tension Calibration bar was set from 0.1 to 1.5. **(B)** Na^{+} imaging micrographs and traces of relative ENG fluorescence intensity (Ft/F0). **(C)** Cl^{-} imaging micrographs and traces of relative MQAE fluorescence intensity (F0/Ft). Mean of ≥ 3 experiments ± SEM. Values marked with asterisks represent significantly different.

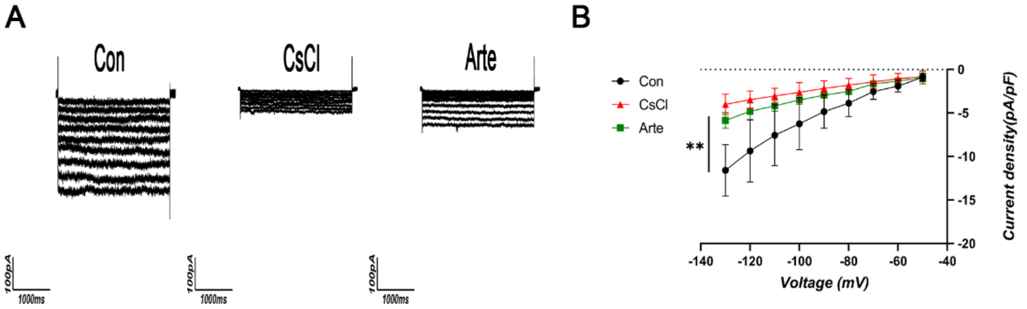

Figure S3. Artemisinin effectively reduced the HCN current. (A) Representative whole-cell currents activated under different treatments. The currents were elicited with 3000-ms voltage steps from –50 mV to –130 mV in 10 mV increments. Artemisinin, Arte; CsCl, pan inhibitor of ion channels. (**B)** The current densities-voltage relationship of HCN currents activated by different treatments.
